# A six years trend analysis of systemic antibiotic consumption in Northwest Ethiopia

**DOI:** 10.1371/journal.pone.0290391

**Published:** 2024-01-31

**Authors:** Asrat Agalu Abejew, Gizachew Yismaw Wubetu, Teferi Gedif Fenta

**Affiliations:** 1 Department of Pharmaceutics and Social Pharmacy, School of Pharmacy, College of Health Sciences, Addis Ababa University, Addis Ababa, Ethiopia; 2 Department of Pharmacy, College of Medicine and Health Sciences, Bahir Dar University, Bahir Dar, Ethiopia; 3 Amhara Public Health Institute, Bahir Dar, Ethiopia; Yekatit 12 Hospital Medical College, ETHIOPIA

## Abstract

**Background:**

Consumption of antibiotics, a major global threat to public health, is perhaps the key driver of antibiotic resistance. Monitoring antibiotic consumption is crucial to tackling antimicrobial resistance. This study assessed antibiotic consumption trends during the last six years in the Bahir Dar branch of the Ethiopian pharmaceutical supply agency (EPSA), Northwest Ethiopia, in 2022.

**Methods:**

Retrospective data were collected in August 2022 based on antibiotic distribution data from the Bahir Dar Brach of EPSA from July 2016 to June 2022. Data were analyzed according to the Anatomic Therapeutic Classification (ATC) developed by the World Health Organization (WHO). We measured antibiotic consumption using a defined daily dose per 1000 inhabitants per day (DIDs) based on the Agency’s catchment population. Descriptive statistics and trend analyses were conducted.

**Results:**

About 30.34 DIDs of antibiotics were consumed during the six years. The consumption of antibiotics decreased by 87.4%, from 6.9 DIDs in 2016/17 to 0.9 DIDs in 2021/22. Based on the WHO AWaRe classification, 23.39 DIDs (77.1%) of the consumed antibiotics were from the Access category. Consumption of Access category antibiotics was decreased by 72.7% (from 5 to 0.5 DIDs) but Watch antibiotics decreased by 54.3% (from 1.8 to 0.4 DIDs). Oral antibiotics accounted for 29.19 DIDs (96.2%) of all consumed systemic antibiotics. The average cost expenditure per DDD for all antibiotics was 54.1 birr/DDD (0.4–482.3 birr/DDD). Only seven antibiotics accounted for DU90% and the cost expenditure per DDD for the DU90% antibiotics ranged from 0.4/DDD for Doxycycline to 232.8 birr/DDD for Piperacillin/tazobactam. Overall, during the last six years, the most commonly used antibiotic was Amoxicillin (10.1 DIDs), followed by Doxycycline (5.3 DIDs) and Ciprofloxacin (3.4 DIDs).

**Conclusion:**

In this study, we found that antibiotic usage was low and continuously declining over time. Minimizing unnecessary antibiotic usage is one possible approach to reduced AMR. However, a shortage of access to important medicines can compromise the quality of treatment and patient outcomes. A prospective study is needed to evaluate the balance of patient outcomes and reduce AMR by optimizing the community consumption of systemic antibiotics.

## Introduction

Consumption of antimicrobials is perhaps the key driver of antibiotic resistance (AMR), a major global threat to public health [1, 2]. The rise in the consumption of last-resort antibiotics is a serious public health concern [2, 3]. Global antibiotic consumption increased by 36% between 2000 and 2010 [2], by 46% between 2000 and 2018 [3], and from 2000 to 2015, global per-capita consumption of watch antibiotics increased by 90.9% [4]. The WHO Access, Watch, and Reserve (AWaRe) antibiotic classification was developed to measure and drive improvements in antibiotic stewardship efforts on global, regional, and national levels [2–4]. This calls for periodic antibiotic consumption evaluation as an important component of AMR prevention and reduction strategies [2, 5, 6]. For this purpose, the WHO developed annually updated guidelines on the Anatomical Therapeutic Chemical (ATC) classification system and Defined Daily Dose (DDD) as a measuring unit for monitoring antibiotic consumption using a standardized population-based metric and research [7].

Low- and middle-income countries (LMICs) are face two challenges: inappropriate antibiotic therapy, lack of access to effective antibiotics, unregulated dispensing, manufacturing of antibiotics with a lack of money to pay for appropriate, high-quality antibiotics, and high rates of AMR and multidrug-resistant bacteria [3, 8, 9]. From 2000 to 2015, the consumption of Watch antibiotics increased by 165.0% in [4]. In LMICs, antibiotic consumption in Sub-Saharan Africa is very low [3] and varies across countries, in Tanzania it was **1**54.51 DIDs from 2010–2016 [10] and 241.41 DIDs from 2017 to 2019 [11], 58.41 DIDs in Ethiopia from 2016 to 2020 [12], 104,791,827 DDD in Uganda from 2017 to 2019 [13], and 19 DIDs in Sierra Leone from 2017 to 2019 [14]. Overall, antibiotic expenditures are increasing in public healthcare institutions [15]. If appropriate intervention is not in place, AMR will have health and social consequences, resulting in increased morbidity and mortality, healthcare costs, and a negative impact on economic growth [16].

In Ethiopia, data on antibiotic consumption using standard measures like DIDs is limited [12, 17, 18], particularly in the study area. On the other hand, the consumption of antibiotics in outpatient departments was reported to be high [17], and in inpatient wards, three out of four patients were prescribed antibiotics [18]. National community-based consumption of systemic antibiotics increased by 16.4% (from 11.02 DID to 12.83 DID) from 2016 to 2020 [12]. Determining antibiotic consumption using a standardized population-based metric, such as DIDs calculated based on import and sales data, and classifying them based on WHO AWaRe can serve as a proxy indicator for antibiotic use at the patient level [7, 19]. This allows the audit of patterns of antibiotic utilization and the identification of problem areas to devise appropriate interventions [7]. Thus, this study was conducted to determine antibiotic consumption using DIDs for six years in Northwest Ethiopia.

## Methods

### Study area and period

An institution-based cross-sectional study was conducted from the EPSA Bahir Dar Branch registers in August 2022 based on drugs distributed from 2016 to June 2022. EPSA is a legal entity established under the Federal Government to assure an uninterrupted supply of pharmaceuticals to the public at an affordable price. The EPSA has 19 branches grouped into seven clusters. EPSA Bahir Dar Brach, located in Bahir Dar City, is one of these Branches serving as outposts to distribute pharmaceuticals, chemical reagents, medical supplies, and equipment to the localities [20]. It serves more than 9 million people in two regions (Amhara National Regional State and Benishangul Gumuz National Regional State) clustered in six Zones (South Gondar, East Gojjam, West Gojjam, Awi Administrative and Metekel Zones, and Bahir Dar City Administration) and 67 woredas. It supplies both public and private health facilities (38 hospitals and 355 health centers) [21]. Since we have used the Ethiopian fiscal year, it should be understood that 2009 = 2016/17, 2010 = 2017/18, 2011 = 2018/19, 2012 = 2019/20, 2013 = 2020/21, 2014 = 2021/22 and for the sake of convenience, we used the next year in the figures, i.e., 2017–2022 is used in the figures.

### Data collection process

The study included all systemic antibiotics procured and distributed and the associated cost expenditure in each year (July 2016 to June 2022) in the EPSA Bahir Dar Branch. The dosage form, strength, the unit of measurement (packs, boxes (number of tablets or capsules), vials, ampoules, MIU, Unit dose, etc.), amount delivered, unit cost and total cost were documented in the registry.

### Outcome measures

Based on the registry, AMC too was used to assign DDDs for each antibiotic based on WHO’s 2019 Anatomical Therapeutic Chemical Classification System (ATC/DDD) and AWaRe classification [7, 19]. It was then standardized into DIDs based on the catchment population [21]. In addition, the number of drugs accounted for 90% of antibiotic consumption (DU90%), and the costs for the DU90% antibiotics and all antibiotics were also calculated as cost per DDD (birr/DDD) was calculated. The DDD/1000 inhabitants/day (DID) was calculated based on the following formula:

$$\frac{\mathrm{DDD}}{1000\;\mathrm{inhabitants}\times \mathrm{day}}=\frac{\mathrm{Amount}\;\mathrm{of}\;\mathrm{drug}\;\mathrm{sold}\;\mathrm{in}\;\mathrm{one}\;\mathrm{year}\;(\mathrm{mg})}{\mathrm{DDD}\;(\mathrm{mg})\times 365\;\mathrm{days}\times \#\mathrm{of}\;\mathrm{inhabitants}}\times 1000\;\mathrm{inhabitants}$$

### Data analysis

Data were cleared, and DDDs and DIDs were calculated for each antibiotic distributed over six years. DDD and DID were calculated separately for antibiotics marketed in fixed-dose combinations (Trimethoprim and Sulfamethoxazole). For antibiotics combined with **β**-Lactamase inhibitors, only the amount of antibiotics was calculated. In addition, based on the DDD, DU90% and cost per unit were calculated. Descriptive statistics and trend analyses were performed. Finally, the results are presented in the tables and figures.

### Operational definitions and definition of terms

- **AWaRe antibiotic classification**: The Access, Watch, and Reserve (AWaRe) classification is a useful tool developed by the WHO to reduce antimicrobial resistance and ensure access to key antibiotics by balancing appropriate access to antibiotics and stewardship use. Access antibiotics indicate the antibiotics of choice for each of the 25 most common infections, which should be available at all times, affordable, and quality-assured. Watch antibiotics include most of the highest-priority, critically important antibiotics for human medicine and veterinary use, which are recommended only for specific, limited indications, whereas reserve antibiotics are those that should only be used as a last resort when all other antibiotics have failed.
- **Defined Daily Dose (DDD):** The assumed average maintenance dose per day for a drug used for its main indication in adults.
- Defined Daily Doses (DDDs) per 1000 inhabitant days per year (DIDs): To standardize the metrics of measurement, the DID **w**as used to measure distributed antibiotics from EPSA, which was calculated as DDDs *1000 days / (catchment population×365 days).
- **The drug utilization 90% (DU90%):** Is an inexpensive, flexible, and simple method for assessing the quality of drug prescribing in routine health care for drugs accounting for 90% of the volume of the prescribed drugs after ranking the drugs used by volume of DDD
- **Cost expenditure for antibiotics:** Shall mean the money spent on purchasing antibiotics.

### Ethical consideration

Ethical Approval was obtained from the ethical review board of Addis Ababa University, the College of Health Sciences, and the School of Pharmacy. A letter of Support for the EPSA was obtained from the Amhara Public Health Institute (APHI). As these were secondary data, informed consent was not obtained. All the information provided was kept confidential and was used only for research purposes.

## Results

### Overall trend and consumption of antibiotics

During the last six years (2016/17 to 2021/22), the Bahir Dar Branch of EPSA distributed a total of 99, 682, 410 DDDs, or 30.34 DIDs (average 5.33 DIDs in year) of antibiotics to various health facilities and sectors under study.

**Fig 1:**
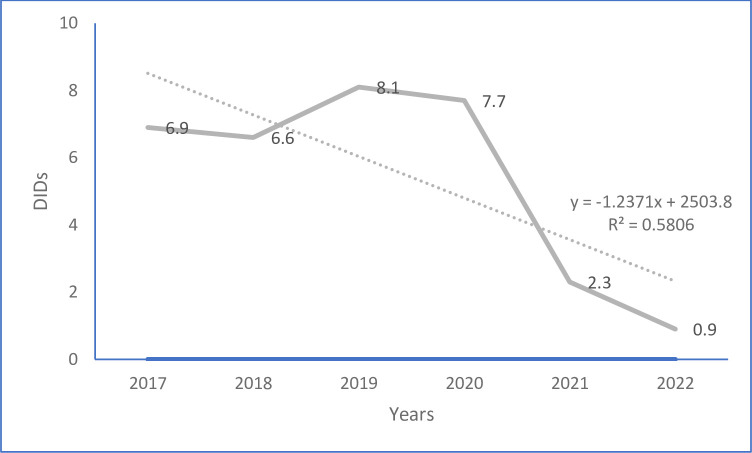
Trends of antibiotic consumption (July 2016- June 2022), Bahir Dar Branch of EPSA, Ethiopia.

From 2016/17 to 2021/22, the overall antibiotic consumption decreased by 87.4% (from 6.9 to 0.9 DIDs). The trend analysis revealed that the overall antibiotic consumption decreased linearly as a function of y = -1.2371x + 2493.9 (R^2^ = 0.5806), and will decrease by 58.1% in each year (Fig 1).

### Antibiotic consumption and trends in different districts/zones

The majority, 7.8 (25.9%) DIDs, were distributed to East Gojjam Zone, followed by West Gojjam Zone, 7.55 (24.9%), and South Gondar Zone, 6.21 (20.5%) (Table 1).

**Table 1:** Distribution of antibiotics in Zones and facilities (July 2016—June 2022), Bahir Dar Brach of EPSA, Ethiopia.

Distribution to		DIDs in each year						
Zones		2016/17	2017/18	2018/19	2019/20	2020/21	2021/22	Total
	East Gojjam Zone	1.73	1.52	1.87	1.95	0.63	0.14	7.85
	West Gojjam Zone	1.76	1.65	1.94	1.70	0.41	0.10	7.55
	South Gondar Zone	1.48	1.19	1.52	1.46	0.43	0.13	6.21
	Awi administrative Zone	0.93	1.03	1.20	1.11	0.37	0.36	5.00
	Bahir Dar City administration	0.69	0.56	0.61	0.56	0.27	0.10	2.79
	Others *	0.12	0.04	0.39	0.33	0.05	0.01	0.94
	Total	6.7	6.0	7.5	7.1	2.2	0.8	30.3

Others*: Central Gondar Zone, West Gondar Zone, Waghimra Administrative Zone, Other Hubs, Metekel Zone

### Trends in consumption by antibiotic class

The consumption trend in each category is almost similar. However, for some classes of antibiotics, it was relatively high in 2017/18 and 2018/19. The most commonly consumed classes of antibiotics were beta-lactam antibiotics, and penicillins (J01C), with 14.9 DIDs, followed by tetracyclines (J01A) and quinolones (J01M), which had 5.3 DIDs and 4.1 DIDs, respectively. Aminoglycosides (J01G) were the least consumed antibiotics, with 0.2 DIDs (Table 2).

**Table 2:** Class of antibiotics consumed (July 2016 –June 2022), Bahir Dar Branch of EPSA, Ethiopia.

Class of antibiotics	Year						
	2016/17	2017/18	2018/19	2019/20	2020/21	2021/22	Total
Beta-lactam antibiotics, penicillins (J01C)	2.918	3.463	3.911	3.098	1.165	0.311	14.866
Tetracyclines (J01A)	1.272	0.558	1.354	2.085	0.076		5.344
Quinolones (J01M)	0.721	0.721	0.842	1.190	0.387	0.209	4.070
Other antimicrobials (J01X)	0.608	0.492	0.808	0.194	0.030	0.137	2.269
Macrolides, lincosamides (J01F)	0.266	0.292	0.357	0.085	0.248	0.072	1.320
Sulfonamides & trimethoprim (J01E)	0.723	0.080	0.030	0.154	0.009	0.071	1.067
Other beta-lactams (J01D)	0.105	0.193	0.205	0.165	0.239	0.046	0.952
Amphenicols (J01B)	0.080	0.091	0.004	0.056			0.232
Aminoglycosides (J01G)	0.024	0.092	0.028	0.078	0.004		0.225
Total	6.716	5.981	7.539	7.105	2.158	0.845	30.34

### Consumption of antibiotics based on WHO AWaRe classification

**Fig 2:**
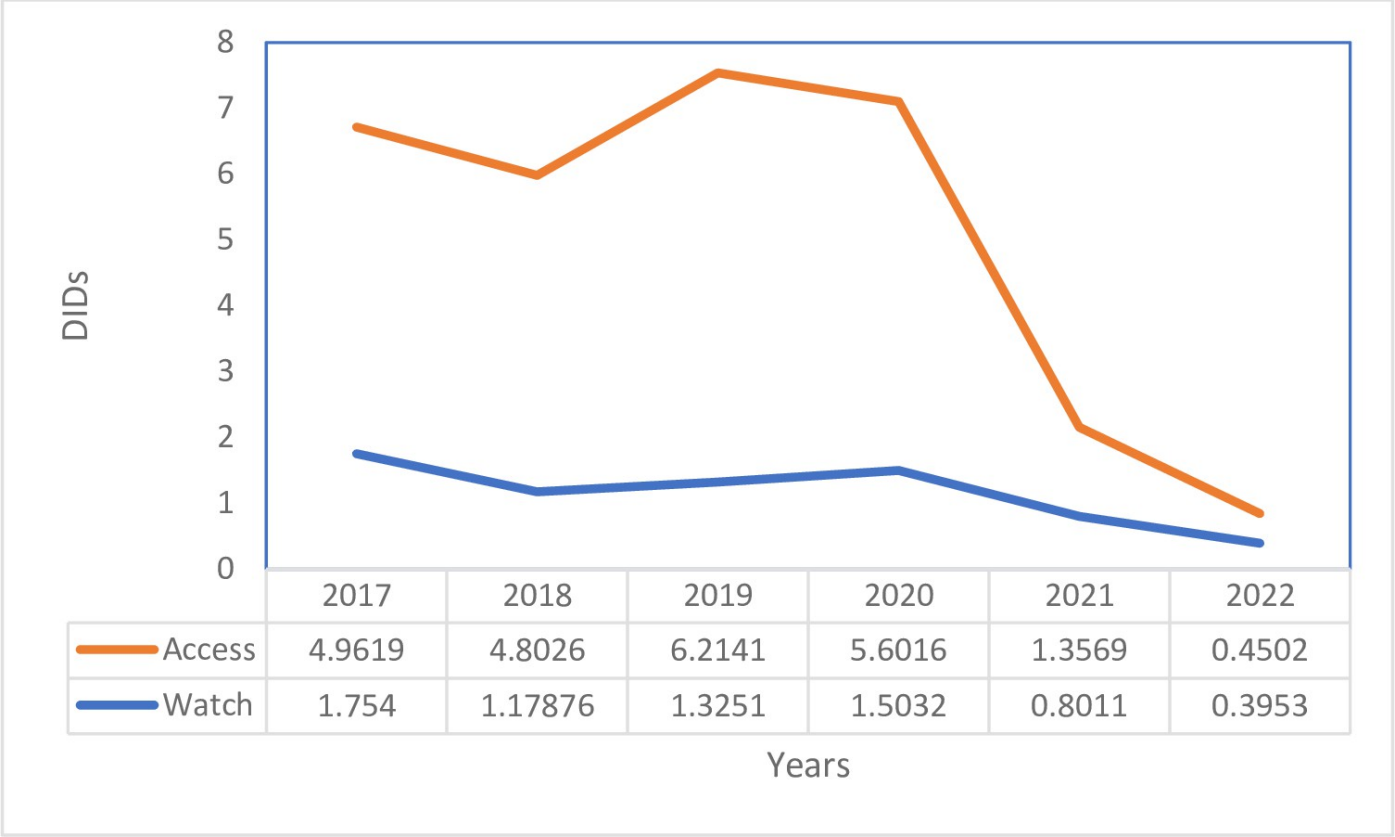
Trends of antibiotic consumption based on WHO AWaRe classification (July 2016- June 2022), Bahir Dar Branch of EPSA, Ethiopia.

Based on the WHO AWaRe classification, 23.39 DIDs (77.1%) of consumed antibiotics were from the Access category, and 6.95 DIDs (22.9%) were from the Watch category. Between 2016/17 and 2021/22, consumption of Access antibiotics decreased by 72.7% (from 5 to 0.5 DIDs) compared with Watch antibiotics, which decreased by 54.3% (from 1.8 to 0.4 DIDs) (Fig 2).

### Consumption of specific antibiotics

Oral antibiotics accounted for 29.19 of total DIDs (96.2%), and parenteral antibiotics accounted for 1.15 DIDs (3.8%). Only seven antibiotics (Amoxicillin, Doxycycline, Ciprofloxacin, Cloxacillin, Metronidazole, Ampicillin, and Piperacillin/tazobactam) accounted for DU90%. Overall, during the last six years, Amoxicillin (10.1 DIDs) was the most commonly consumed antibiotic, followed by Doxycycline (5.3 DIDs), Ciprofloxacin (3.4 DIDs), and Cloxacillin (3.06 DIDs). Compared with baseline consumption, Azithromycin, Ceftazidime, and Vancomycin were slightly increased, but for others, it either decreased or remained constant (Table 3).

**Table 3:** Consumption of each antibiotic (July 2016 –June 2022), Bahir Dar Branch of EPSA, Ethiopia.

Antibiotics	DID in each year						
	2016/17	2017/18	2018/19	2019/20	2020/21	2021/22	Total
Amoxicillin	1.9947	2.3467	3.0016	1.9268	0.6914	0.1216	10.0827
Amoxicillin /clavulanic	0.0746	0.0705	0.1034	0.0698	0.2262	0.0228	0.5673
Ampicillin	0.3166	0.4576	0.2717	0.0592	0.0099	0.0065	1.1214
Ampicillin/sulbactam	0.0000	0.0000	0.0000	0.0001	0.0001	0.0000	0.0003
Azithromycin	0.0001	0.0023	0.1369	0.0000	0.0526	0.0668	0.2587
Cefepime	0.0000	0.0000	0.0000	0.0001	0.0304	0.0000	0.0306
Cefixime	0.0000	0.0001	0.0000	0.0002	0.0007	0.0000	0.0011
Cefotaxime	0.0000		0.0020	0.0000	0.0140	0.0000	0.0160
Ceftazidime	0.0000	0.0001	0.0000	0.0000	0.0001	0.0000	0.0003
Ceftriaxone	0.0008	0.0010	0.0002	0.0019	0.0037	0.0019	0.0094
Cefuroxime	0.0432	0.0862	0.0909	0.0719	0.1034	0.0401	0.4356
Cephalexin		0.0001					0.0001
Cefazoline	0.0606	0.1051	0.1115	0.0907	0.0863	0.0044	0.4586
Chloramphenicol	0.0796	0.0914	0.0044	0.0563		0.0000	0.2317
Ciprofloxacin	0.5722	0.6378	0.6868	0.9798	0.3243	0.1996	3.4006
Clarithromycin	0.1538	0.2813	0.0649	0.0643	0.1953	0.0030	0.7628
Clindamycin	0.0006	0.0019	0.0010	0.0029		0.0000	0.0063
Cloxacillin	0.5282	0.5797	0.5249	1.0364	0.2270	0.1603	3.0564
Doxycycline	1.2670	0.5175	1.3500	2.0849	0.0758	0.0000	5.2953
Erythromycin	0.1118	0.0062	0.1543	0.0178	0.0000	0.0021	0.2922
Gentamicin	0.0235	0.0918	0.0276	0.0776	0.0043	0.0000	0.2248
Meropenem	0.0000	0.00006				0.0000	0.0000
Metronidazole	0.6066	0.4913	0.8029	0.1908	0.0248	0.1343	2.2506
Norfloxacin	0.1491	0.0830	0.1550	0.2104	0.0628	0.0091	0.6695
Penicillin G	0.0038	0.0086	0.0099	0.0054	0.0101	0.0000	0.0378
Piperacillin/Tazobactam	0.7230	0.0802	0.0303	0.1542	0.0091	0.0707	1.0674
SXT	0.0049	0.0400	0.0041			0.0000	0.0491
Tetracycline	0.0012	0.0004	0.0011	0.0007	0.0010	0.0003	0.0045
Vancomycin	0.0000	0.0005	0.0038	0.0026	0.0047	0.0020	0.0136
Total	**6.7161**	**5.9806**	**7.5390**	**7.1050**	**2.1580**	**0.8455**	**30.3442**

SXT: Sulfamethoxazole- trimethoprim

### Cost expenditure for antibiotics

**Fig 3:**
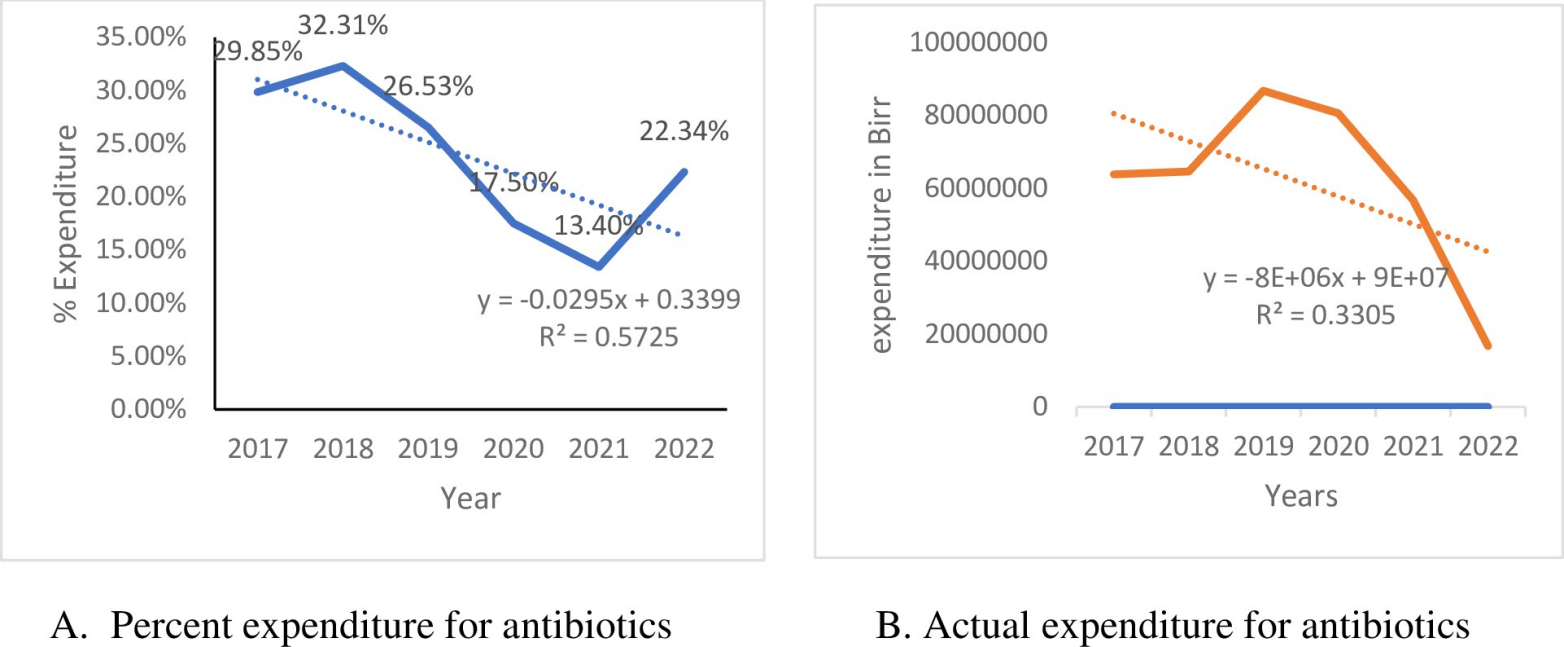
Trends in antibiotic expenditure (July 2016- June 2022), Bahir Dar Branch of EPSA, Ethiopia. A. Precent expenditure for antibiotics, B. Actual expenditure for antibiotics.

The overall cost expenditure for purchasing antibiotics was $7,281,680.907 (385, 200, 920 Ethiopian Birr), which increased from 29.85% in 2016/17 to 32.31% in 2017/18, then decreased to 26.53% in 2018/19, 17.5% in 2019/20, and 13.4% in 2020/21. The percentage cost expenditure on antibiotics based on the total cost expenditure for drugs and the actual cost expenditure for antibiotics decreased linearly from time to time as a function of **y = -0.0295x+0.3399** (R^2^ = 0.5725) and **y = -8E+06x + 9E+**07 (R^2^ = **0.3305**) as shown in Fig 3A and 3B, respectively (Fig 3).

About 74.4% of the total expenditures for purchasing antibiotics were used for purchasing Access antibiotics, and only 25.6% were for purchasing Watch antibiotics. From 2016/17 to 2021/22, the total expenditure for purchasing antibiotics decreased by 64%. Similarly, the cost expenditure for Access and Watch antibiotics decreased by 72.3% and 33.6%, respectively. Of the total antibiotic cost expenditures, 26.4% were used to purchase Amoxicillin, followed by Cloxacillin (13.6%), Metronidazole (10.5%), Ceftriaxone (9.6%), and Amoxicillin/clavulanic (6.1%). Only about 62.7% of the expenditure was used to purchase DU90% antibiotics.

The average expenditure per DDD for all antibiotics was 54.1 birr/DDD (0.4–482.3 birr/DDD). The highest cost expenditure per DDD was for Cefotaxime (91.1), Ceftazidime (120.8), Cefepime (133.5), Vancomycin (160.4), Meropenem (214.0), Piperacillin/tazobactam (232.8), and Ampicillin/sulbactam (482.3) in increasing order. The cost per DDD for the DU90% antibiotics was Amoxicillin (2.8 birr/DDD), Doxycycline (0.4/DDD), Ciprofloxacin (1.6 birr/DDD), Cloxacillin (4.8 birr/DDD), Metronidazole (2.9 birr/DDD), Ampicillin (4.9 birr/DDD), and Piperacillin/tazobactam (232.8 birr/DDD). The trend and overall cost expenditure per DDD are presented in (Table 4).

**Table 4:** Trends in cost expenditure per DDD of antibiotics (July 2016-June 2022), Bahir Dar Branch of EPSA, Ethiopia.

Antibiotic	Cost expenditure /DDD						
	2016/17	2017/18	2018/19	2019/20	2020/21	2021/22	Total
Amoxicillin	2.7	2.6	2.9	3.0	2.9	3.3	2.8
Amoxicillin /clavulanic	12.9	9.2	8.7	15.6	12.0	7.7	11.4
Ampicillin	3.8	3.2	6.2	11.8	33.7	21.9	4.9
Ampicillin/sulbactam	280.1	232.4	1062.1	498.4	510.3		482.3
Azithromycin	19.3	3.3	2.5		8.0	2.0	3.5
Cefazoline		81.1		81.1	0.1		0.5
Cefepime		124.4	108.4	108.4	144.7		133.5
Cefixime			19.2	19.8	6.8		8.4
Cefotaxime	64.2	68.5	95.2	73.1	128.0	0.0	91.1
Ceftazidime	225.8	88.2	117.4	92.9	140.7	83.1	120.8
Ceftriaxone	23.6	17.3	27.5	22.3	25.0	24.1	23.4
Cefuroxime		33.7					33.7
Cephalexin	6.2	6.0	9.6	8.3	11.8	14.3	8.6
Chloramphenicol	11.1	5.2	33.3	22.3			10.2
Ciprofloxacin	1.7	1.5	1.1	1.6	2.0	2.3	1.6
Clarithromycin	3.9	3.8	6.0	4.3	5.7		4.5
Clindamycin	15.8	12.2	23.4	18.1			16.9
Cloxacillin	3.2	3.1	6.1	4.8	9.7	5.4	4.8
Doxycycline	0.4	0.4	0.4	0.3	0.4		0.4
Erythromycin	5.6	8.4	4.8	12.4		12.2	5.8
Gentamicin	6.2	5.0	1.3	5.1	0.7		4.6
Meropenem		210.8	252.5				214.0
Metronidazole	1.4	2.5	2.5	5.0	11.9	36.2	2.9
Norfloxacin	1.7	1.5	1.7	1.7	1.9		1.7
Penicillin G	42.8	21.2	26.1	29.8	27.1		27.3
Piperacillin/Tazobactam		232.8					232.8
Sulphathiazole	3.6	5.0	13.3	5.6	31.0	2.8	4.4
Tetracycline	4.0	1.2	1.3				1.5
Trimethoprim	2.5	5.0	8.7	5.6	33.5	13.1	3.9
Vancomycin	157.0	135.3	147.5	171.3	176.7	143.2	160.4
Total	899.5	1324.8	1989.7	1222.6	1324.6	371.6	1622.6

## Discussion

This study attempted to quantify antibiotic consumption based on available data from the Bahir Dar Branch of EPSA, Northwestern Ethiopia, from July 2016 to June 2022. In general, the overall consumption of antibiotics was 30.34 DIDs, which decreased by 87.4% (from 6.9 to 0.9 DIDs) from July 2016 to June 2022. From 30.34 DIDs, 14.9 DIDs accounted for beta-lactam antibiotics, and penicillins (J01C), and 23.39 DIDs were for Access antibiotics. The overall consumption of antibiotics decreased by 87.4%, and consumption of Access antibiotics decreased by 72.7%. About 29.19 DIDs (96.2%) of systemic antibiotics were administered orally. The total cost expenditure for antibiotics accounted for $7,281,680.907, of which 74.4% was used for purchasing access antibiotics.

Antibiotic consumption is a major driver of AMR [2]. Variations in AMR across different countries are attributable to the difference in volume and pattern of antibiotic consumption [2, 22, 23]. In this study, total antibiotic consumption from July 2016 to June 2022 was 30.34 DIDs. This is low than the national consumption of antibiotics from 2016 to 2020 in Ethiopia, which was 58.95 DIDs [12], and it was extremely low compared with a study in Tanzania, which reported consumption of 242.41 (80.80 ± 39.35) of antibiotics from 2017 to 2019 [13]. Compared with the estimated global antibiotic consumption rate of 14.3 DIDs from 2000 to 2018 [3] and the total consumption of antibiotics in Sierra Leone from 2017 to 2019, which was 19 DIDs [14], the consumption of antibiotics in the current study was high. The current study’s low level of antibiotic consumption might be linked to budget shifts to save lives due to increased internal displacements in the region due to instabilities and inflated global market dynamics due to the COVID-19 pandemic. However, the decline in the consumption of antibiotics from government sources could have resulted in an alternative source of antibiotic supply, which could affect affordability in the community, probably fueling the emergence and spread of AMR.

The current study reported an 87.4% (from 6.9 to 0.9 DIDs) decrease in antibiotic consumption from July 2016 to June 2022. This was in contrast to national consumption from 2016 to 2020, which increased by 16.4% (from 11.02 DID to 12.83) [12], a global report, which increased by 46% (92–1005 DIDs) from 2000 to 2018 [3], a Tanzanian study, which increased by 610.8% (6.78–48.19 DIDs) from 2010 to 2016 [10], and an Indian study reported 22% increase in antibiotic consumption from 2008 to 2012 [24]. Similar to our finding, a decrease in the consumption of antibiotics reported in a recent study in Tanzania, declined by 29.33% (136.41 to 54.98 DIDs) from 2017 to 2019 [11], and in another recent study from India, and then decline by 3.6% between 2011–2019 [25]. The decline was 3.6% between 2011 and 2019 [25]. The highest decrease in antibiotic consumption in the current study from 2019/20 might be directly related to the COVID-19 pandemic, as was evident in the global antimicrobial consumption level decrease of 18.7% worldwide from April to August 2020 compared with the previous year, 2019 [26]. The political instabilities in the country that could have shifted attention and health priority to survival issues, resulting in a reduced supply of antibiotics. The difference in trends in consumption [27–29] in the current study may be attributed to the decrease in cost expenditure for antibiotics and inflation in the cost of antibiotics, indicating that the possible reason for the decrease in antibiotic consumption is related to the decrease in expenditure, as well as inflation in drug cost and currency.

This study accounted for 14.9 DIDs out of 30.3 DIDs for **β**-lactam antibiotics and penicillins (J01C). This is similar to national antibiotic consumption in Ethiopia [12]. Similar results have been reported in Bhutan, where the penicillin group of beta-lactam antibacterials (J01C) was the most commonly used subgroup [30]. This was due to the overwhelming use of Amoxicillin and other penicillins in healthcare facilities because they are the antibiotics of choice for many indications in inpatient and outpatient settings. The WHO recommends that the consumption of access antibiotics should make up at least 60% of national consumption [19], justifying the overwhelming use of penicillin groups in the current study.

In the current study, the consumption of access antibiotics was 77.1%, and watch antibiotics accounted for 22.9%. Although consumption of access antibiotics was in agreement with WHO’s minimum 60% recommendation [20], the value is high compared with a study in Sierra Leone [14] and China [27], which reported 65% and 42% of antibiotic consumption in the Access category, respectively. However, the consumption of Watch antibiotics (31%), was relatively higher in Sierra Leone [14] than in the current study. The current study, similar to Sierra Leone’s case [14], didn’t report the consumption of reserve antibiotics. The difference might be due to differences in government policy and accessibility of antibiotics.

Similar to the overall trend in antibiotic consumption, in the current study, the consumption of Access antibiotics decreased by 72.7% (from 5 to 0.5 DIDs), and the consumption of Watch antibiotics decreased by 54.3% (from 1.8 to 0.4 DIDs). This was in contrast to the WHO’s assessment from 2000 to 2015, which showed consumption of Watch antibiotics increased by 90.9% (from 3.3 to 6.3 DIDs) and 26.2% (from 8.4 to 10.6 DIDs) in Access antibiotics [4]. In India, the consumption of Access antibiotics decreased by only 13.1% [24], which is lower than that in the current study.

The current study revealed 29.19 DIDs (96.2%) for consumption of oral antibiotics and 1.15 DIDs (3.8%) of parenteral antibiotics. This is in agreement with a study in Sierra Leone, which showed 98.4% oral antibiotic consumption and 1.6% parenteral antibiotics consumption [14]. The consumption of oral antibiotic was higher in the current study than in China, which reported less than 70% oral antibiotic consumption and 31%–36% of parenteral total antibiotic consumption [27]. In Uganda, the oral route of administration accounted for 59% and the parenteral route accounted for 41% [13], which was also different from the current study.

Using the current exchange rate of (52.9 as of 25/11/2022), the current study’s total antibiotic expenditure was $7,281,680.907 (385,200,920 Ethiopian Birr). This was lower than the $56.0 billion expenditure on purchasing antibiotics over the six-year (between 2010 and 2015) in healthcare settings in the United States [31]. It was also low compared with the overall antibiotic expenditure in public healthcare institutions in Shandong Province in China from 2012 to 2016, which accounted for $717 million, a 56% increase from the baseline [15]; in the current study, the total expenditure for purchasing antibiotics decreased by 64%. In the current study, 74.4% of the total antibiotic expenditure was used to purchase access antibiotics, and only 25.6% was for purchasing antibiotics in the Watch category, which was contrary to another study in China, which showed 55.57% expenditure on the Watch group and 9.91% for Access group antibiotics [29].

DU90% is an inexpensive, flexible, and simple method for assessing the quality of antibiotic prescribing in routine healthcare [32]. Studies on antibiotic consumption using the DU90% methodology has also been conducted at community level [33]. In the current study, only seven antibiotics (Amoxicillin, Doxycycline, Ciprofloxacin, Cloxacillin, Metronidazole, Ampicillin, and Piperacillin/tazobactam) constituted the DU90%. This is different from national antibiotic consumption, which reported that nine antibiotics (Amoxicillin, Doxycycline, Ciprofloxacin, Cloxacillin, Metronidazole, Trimethoprim, Sulfamethoxazole, Ampicillin, and Norfloxacin) constituted DU90% of the whole consumption [12]. However, the cost expenditure of the seven antibiotics in the current study accounted for only 62.7% of the cost used to purchase DU90% of drugs. This was different from the 80.8% total cost expenditure for DU90% antibiotics compared with the 19.2% for the remaining 10% [32]. The highest cost per DDD was among antibiotics in the 10%. A substantial amount of money can be saved without compromising the quality of care by using recommended and commonly utilized antibiotics [34].

Determining antibiotic consumption based on procurement or sales data serves as an overall proxy indicator for actual exposure in the community [35]. Although reducing unnecessary antibiotics, improving access, implementing and measuring stewardship programs, and tracking and using data are possible advanced solutions to tackle AMR [36], the positive impact of antibiotics on health is frequently threatened by increasing levels of antimicrobial resistance (AMR) and hampered by a lack of access to essential antibiotics [3]. A continuously reduced supply of antibiotics may have many implications for healthcare facilities in the catchment area. According to Namomsa G., among the community-based health insurance (CBHI) users, only 60% of the members reported getting prescribed medicines after being prescribed for treatment. The absence of antibiotics in health facilities is an implication for poor service provision, resulting in a reduced enrollment rate for CBHI [37]. Addressing lack of access and inappropriate antibiotic use is crucial for tackling the growing threat of antimicrobial resistance and ensuring fair and equitable access to essential medicines for all [3]. The aftereffects of decreased antibiotic supply have multiple far-reaching negative consequences, ranging from a lack of availability in public health facilities resulting in suboptimal or broad-spectrum antibiotic use or necessitating the use of reserve antibiotics to treat common infections to increased healthcare costs, and eventually, a rise in the number of drug-resistant infections. Thus, identifying the reasons for shortages of antibiotic supply and proposing solutions to address the issue is the first step in the complex process of changing antibiotic supply-related problems.

The strength of the current study is documenting the community-based annual consumption of systemic antibiotics in the regional hub EPSA over six years using standard methodology and applying DID to estimate the general population’s exposure to antibiotics in the catchment area. It also analyzes the trend in antibiotic consumption and expenditure, including the total budget allocated for antibiotics. Nevertheless, there might be limitations arising from the organizational structure of the agency and the nature of secondary data. It will not necessarily show the captured seasonal variations in the community-based consumption of antibiotics in the area. It didn’t conduct validation of antibiotic distribution and, thus, will not show actual patient-level use of antibiotics in the area by simply collecting available resources on drug distribution and extracting antibiotics from them. It also didn’t take into account the possibility of unrecorded drugs in the Hub.

In conclusion, antibiotic consumption was very low in Northwest Ethiopia. Antibiotic consumption, percent allocation from total expenditures to antibiotic procurement, and actual expenditure for antibiotic procurement decreased linearly during the last six years. Consumption of Access category and oral antibiotics was very good, although Access antibiotics decreased from time to time in line with total antibiotic consumption. Seven antibiotics constituted DU90%, and less than 2/3 of the cost was used on DU90% antibiotics. Since EPSA is the sole public supplier in the area, the finding directly implies a reduced supply of antibiotics at the institutional level which could in turn affect the quality of care and patient stratification specifically patients on CBHI may suffer a lot from lack of antibiotics. Antibiotic resistance is a multifactorial problem resulting from multisectoral failures to use antibiotics and enforce stewardship measures appropriately. This calls for a prospective mixed-methods study to understand the spiral of antibiotic usage and resistance which might help to assure a continuous supply of key antibiotics while optimizing the use and reducing unnecessary prescribing practices that fuel AMR.

## Supporting information

S1 Data Raw data on antibiotics. (XLSX)
